# A randomized controlled trial of metformin on left ventricular hypertrophy in patients with coronary artery disease without diabetes: the MET-REMODEL trial

**DOI:** 10.1093/eurheartj/ehz203

**Published:** 2019-04-17

**Authors:** Mohapradeep Mohan, Shaween Al-Talabany, Angela McKinnie, Ify R Mordi, Jagdeep S S Singh, Stephen J Gandy, Fatima Baig, Muhammad S Hussain, U Bhalraam, Faisel Khan, Anna-Maria Choy, Shona Matthew, John Graeme Houston, Allan D Struthers, Jacob George, Chim C Lang

**Affiliations:** 1 Division of Molecular and Clinical Medicine, School of Medicine, Ninewells Hospital and Medical School, University of Dundee, Dundee DD1 9SY, UK; 2 NHS Tayside Clinical Radiology, Ninewells Hospital & Medical School, Dundee, DD1 9SY, UK; 3 Department of Medical Physics, NHS Tayside, Ninewells Hospital & Medical School, Dundee, DD1 9SY, UK

**Keywords:** Coronary artery disease, Left ventricular mass, Metformin, Pre-diabetes, Insulin resistance, Oxidative stress

## Abstract

**Aim:**

We tested the hypothesis that metformin may regress left ventricular hypertrophy (LVH) in patients who have coronary artery disease (CAD), with insulin resistance (IR) and/or pre-diabetes.

**Methods and results:**

We randomly assigned 68 patients (mean age 65 ± 8 years) without diabetes who have CAD with IR and/or pre-diabetes to receive either metformin XL (2000 mg daily dose) or placebo for 12 months. Primary endpoint was change in left ventricular mass indexed to height^1.7^ (LVMI), assessed by magnetic resonance imaging. In the modified intention-to-treat analysis (*n* = 63), metformin treatment significantly reduced LVMI compared with placebo group (absolute mean difference −1.37 (95% confidence interval: −2.63 to −0.12, *P* = 0.033). Metformin also significantly reduced other secondary study endpoints such as: LVM (*P* = 0.032), body weight (*P* = 0.001), subcutaneous adipose tissue (*P* = 0.024), office systolic blood pressure (BP, *P* = 0.022) and concentration of thiobarbituric acid reactive substances, a biomarker for oxidative stress (*P* = 0.04). The glycated haemoglobin A1C concentration and fasting IR index did not differ between study groups at the end of the study.

**Conclusion:**

Metformin treatment significantly reduced LVMI, LVM, office systolic BP, body weight, and oxidative stress. Although LVH is a good surrogate marker of cardiovascular (CV) outcome, conclusive evidence for the cardio-protective role of metformin is required from large CV outcomes trials.

**Figure:**
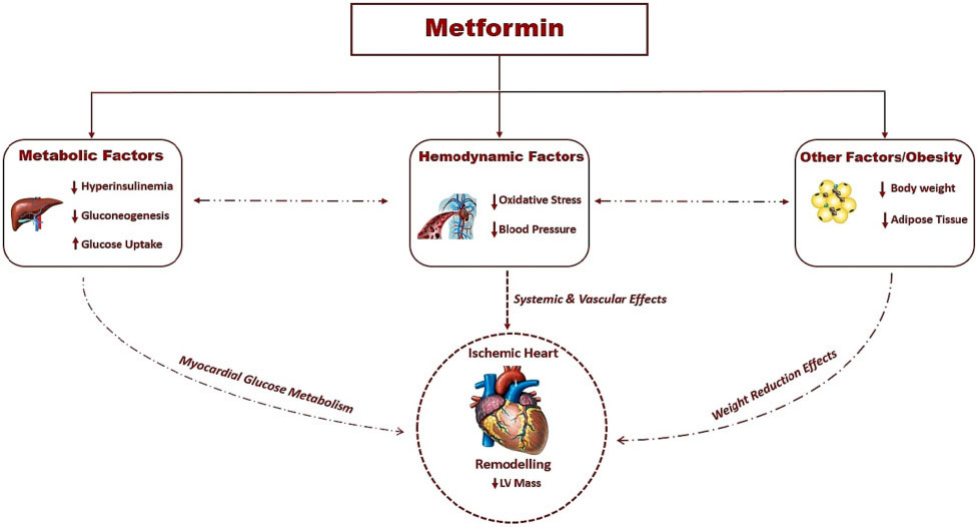

## Introduction

Left ventricular hypertrophy (LVH) is an independent predictor of mortality and is highly prevalent in patients with ischaemic heart disease, even in the absence of hypertension.^1^^,^^2^ Left ventricular hypertrophy is a common finding in approximately one-third of patients with coronary artery disease (CAD).^3^ Importantly, LVH is one of the most powerful prognostic factor in CAD, after age and coronary disease severity with a prognostic importance equivalent to that of left ventricular (LV) ejection fraction.^3^^,^^4^ Regression of LVH can reduce the incidence of major cardiovascular (CV) events irrespective of blood pressure (BP) changes.^5–7^ However, controlling BP is only partially effective as LVH persists in 20% of hypertensive patients who attain target BP.^8^ Therefore, additional strategies are required.

Other than BP, insulin resistance (IR) and central obesity are implicated in the development of LVH. Dysglycaemia is very common in patients with CAD and is linked to IR.^9^ Large studies have reported a significant positive relationship between IR and LVH.^10–12^ Specifically, central obesity has been associated with IR, hypertension, and LVH.^13^ Importantly, non-diabetic dysglycaemia (pre-diabetes) *per se* is associated with substantial CV risk that is now recognized by clinical practice guidelines.^14^

Metformin—an anti-diabetic drug, has been shown to improve insulin sensitivity and reduce IR.^15^ In a meta-analysis of randomized controlled trials (RCTs), Salpeter *et al.*^16^ reported a reduction of weight and calculated IR in metformin users. Metformin has multiple modes of actions involving both AMP-activated protein kinase (AMPK)-dependent and AMPK-independent mechanisms that may be implicated in cardiac hypertrophy.^17^ In this respect, metformin has been shown to reduce cardiac hypertrophy in different animal models of hypertrophy.^18^ Observational studies have also reported CV benefits in metformin users^19^ especially in patients with type 2 diabetes mellitus (T2DM) and heart failure.^20^ For these reasons, there is now much interest in the repurposing of metformin for CV diseases.^21^

In this ‘proof of concept’ RCT, we hypothesized that metformin would cause regression of LVH in patients without T2DM, with CAD and dysglycaemia identified by IR and/or pre-diabetes.

## Methods

### Study design

**Figure 1:**
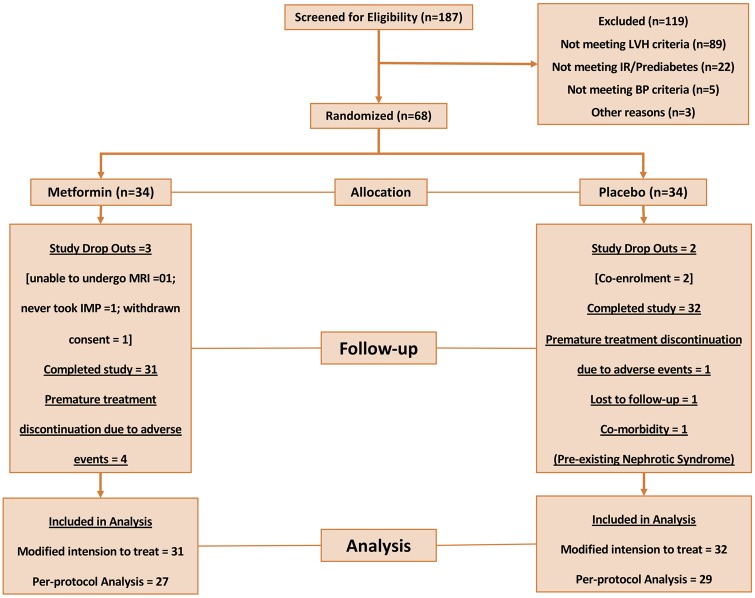
Trial consort diagram.

The MET-REMODEL study (NCT02226510) was a single centre, double-blind, placebo-controlled trial designed to evaluate the efficacy of a 12-month Metformin XL (prolonged release) 1000 mg twice daily treatment on LVH in non-diabetic, participants identified to have CAD, LVH, and IR and/or pre-diabetes. *Figure 1* shows the MET-REMODEL trial consort type flow diagram and Supplementary material online, *Figure S1* shows the study design. The study was approved by the East of Scotland Research Ethical Committee (14/ES/1061 IRAS) and all patients provided written informed consent.

### Study participants

The study population included 68 patients recruited between April 2015 and September 2016 from Tayside, Scotland, using research databases, hospital records, and local general practices. Participating patients were aged 18–85 years with documented CAD (previous myocardial infarction/unstable angina and/or previous revascularization by either percutaneous coronary intervention or coronary artery bypass graft surgery) and without diabetes (ascertained by clinical history extracted from medical notes and screening HbA1c measurement of ≥ 48 mmol/mol). Patients were also required to have IR [IR defined using fasting insulin resistance index (FIRI ≥2.7) measured by an empirical FIRI, consisting of the product of plasma insulin and glucose: FIRI = fasting glucose × fasting insulin/25] AND/OR American Diabetes Association defined pre-diabetes: glycated haemoglobin (HbA1c) ≥39 mmol/mol and <48 mmol/mol.^22^^,^^23^ Patients with hypertension were not excluded from the study but their prevailing BP had to be in the normotensive range with a clinic BP ≤140/85 mmHg (mean value of three measurements performed at 5 min intervals on same arm). Detailed study exclusion criteria are described in Section A in the Supplementary material online. Presence of LVH was based on published sex specific normal values of LV mass allometrically scaled to height (LV mass/height1^.7^ >95th percentile) ascertained by screening echocardiography (males >81 g/h1.7, females >60 g/h1.7).^24^ Given the high prevalence of obesity (BMI ≥ 30 kg/m^2^) in these study patients, we determined LVMI by LVM/height1^.7^, which has been shown to be a sensitive method to assess LVMI.^24^

Participants who met the eligibility criteria were randomly assigned to receive either metformin XL 500 mg twice daily or matching placebo in a double-blind fashion (randomization protocol described in Section B in the Supplementary material online) for 2 weeks. If this was tolerated, the dosage was increased to 1000 mg twice daily for a further 11 months. If the higher dose could not be tolerated, it was reduced to 1000 mg/day, or stopped altogether if this lower dosage was not tolerated.

### Magnetic resonance imaging

Images at baseline and at 12 months were acquired on a 3.0T Tesla magnetic resonance imaging (MRI) scanner (Magnetom Trio-PrismaFIT, Siemens, Erlangen, Germany) using spine matrix and body array matrix RF coils. The cardiac MRI (CMR) and abdominal MRI protocols used are described in Section C in the Supplementary material online.

### Flow-mediated dilatation

Endothelial function was assessed using a Sequoia 512 (Siemens, Camberley, England) ultrasound machine with an 8 MHz linear array probe to measure flow-mediated dilatation (FMD) of the brachial artery in response to hyperaemia at baseline visit and at 12 months, according to the standard guidelines as described by Coretti *et al.*^25^

### Laboratory investigations

Routine biochemical and haematological investigations were measured at all study visits as safety parameters. Fasting plasma glucose, HbA1c, fasting insulin, biomarkers of interest such as interleukin-6 (IL-6), Soluble ST2, N-terminal pro-brain natriuretic peptide (NTproBNP) and thiobarbituric acid reactive substances (TBARs) were measured at baseline and final visit.

Vital signs (office BP, heart rate, and weight) were assessed at every study visit. Adverse events (AEs) and serious adverse events (SAEs) were patient reported.

### Study endpoints

The primary endpoint was to determine whether metformin induces regression of LVMI assessed by CMR. The secondary endpoints were changes in LV ejection fraction, mass, and volumes; abdominal obesity assessed by MRI, glycaemic parameters, endothelial function, and blood biomarkers.

### Power calculation

The sample size was based on changes in LVM, using published data from a previous LVH regression study with CMR (5 g change, SD 2.8) in a similar cohort of patients.^26^ For an 80% power at a 5% significance level (*α* = 0.05), to detect a similar change in LVM, we will require 29 subjects per group in our study. We enrolled 34 patients per group to allow a 15% drop out rate.

### Statistical analysis

The primary outcome comparison was based on modified intention-to-treat (mITT) analysis, i.e. all participants who had baseline measurements and took at least one dose of investigational medicinal product were analysed as part of the group to which they were randomized. Missing post-baseline values were imputed using the baseline observation carried forward (BCOF—all the variables for two patients in the placebo arm) approach. However, to provide a true estimate of the efficacy of intervention, a per-protocol analysis was also performed. The comparison between intervention and placebo groups was compared using independent samples *t*-tests for continuous variables and χ^2^ for dichotomous variables. Continuous variables with normal distribution are presented as mean (SD). Non-normally distributed data were presented as medians alongside their inter-quartile ranges. They subsequently underwent log transformation to achieve normality. Additionally, we performed a sensitivity analysis using analysis of covariance (ANCOVA) model to evaluate the robustness of treatment with change in LVMI, LVM and treatment as fixed effects, and age, sex, and baseline values for LVMI/LMI, body weight, systolic blood pressure (SBP), diastolic blood pressure (DBP), prescription of angiotensin-converting enzyme inhibitor, angiotensin II receptor blockers, and beta-blocker as covariates. A *P*-value <0.05 was considered significant. Data were analysed using SPSS 22.0 (IBM Corp, Armonk, NY, USA).

## Results

### Recruitment and follow-up

Of the 187 participants who were screened, 68 subjects (*Figure 1*) fulfilled all the study criteria and were randomly allocated to receive either metformin (*N* = 34) or placebo (*N* = 34). Five patients were excluded from all study analysis prior to unblinding: no baseline MRI measurements due to claustrophobia (*N* = 1); comorbidity—incidental finding of abdominal aortic aneurysm on MRI that required immediate medical attention prior to starting on study drug (*N* = 1), withdrawn consent (*N* = 1) and protocol breach (*N* = 2; co-enrolment). Seven patients discontinued study medications: premature treatment discontinuation due to AEs (*N* = 5); lost to follow-up (*N* = 1) and pre-existing comorbidity (*N* = 1) however, they were included in our mITT analysis. Sixty-three patients in total completed the study (*N* = 31 in metformin group; *N* = 32 in placebo group) as per the study protocol and were included for analysis in the mITT arm of the study and 56 patients (*N* = 27 in metformin group; *N* = 29 in placebo group) in the per-protocol population.

### Patient characteristics

Baseline characteristics of study population at randomization are shown in *Table 1*. When compared with the placebo group, patients in the metformin group had somewhat higher fasting IR index, absolute LV mass measurements, weighed more, had more frequent positive exercise tolerance testing and use of calcium-channel blockers, although these differences were not statistically significant.

**Table 1:** Baseline characteristics of study population

Variable	All patients (*n* = 63)	Metformin (*n* = 31)	Placebo (*n* = 32)	*P*-value
Demographics				
Age, in years	64.6 ± 8.4	64.5 ± 8.9	64.6 ± 8.0	0.971
Sex (% of males)	47 (75)	26 (84)	21 (66)	0.096
Ex-smokers (%)	3 (5)	16 (52)	17 (53)	0.993
Current smokers (%)	8 (13)	4 (13)	4 (13)	
Alcohol consumption (%)	41 (65)	19 (61)	22 (69)	0.430
Weight, kg	90.3 ± 12.9	93.3 ± 12.9	87.3 ± 12.3	0.074
Body mass index, kg/m^2^	32.0 ± 3.5	32.2 ± 3.4	31.9 ± 3.6	0.419
Systolic blood pressure, mmHg	130.6 ± 10.9	130.8 ± 10.7	130.5 ± 11.2	0.903
Diastolic blood pressure, mmHg	75.8 ± 7.9	75.3 ± 8.0	76.4 ± 7.9	0.591
Pre-diabetes (%)	50 (79)	23(74)	27(84)	0.326
Insulin resistant (%)	44(70)	25(81)	19(59)	0.096
Pre-diabetes + insulin resistance (%)	32 (51)	17 (55)	15 (47)	0.535
Heart rate, b.p.m.	57.7± 9.1	57.6 ± 9.9	57.8 ± 8.4	0.953
MRI study assessments				
Absolute LVM, g	114.8 ± 24.8	120.7 ± 20.3	109.1 ± 27.6	0.064
LVMI (height^1.7^)	47.3 ± 8.1	48.7 ± 6.5	46.0 ± 9.3	0.197
Subcutaneous adipose tissue, cm^3^	3332.7 ± 1044.1	3371.6 ± 1037.0	3301.6 ± 1090.3	0.897
Visceral adipose tissue, cm^3^	2422.6 ± 878.5	2407.1 ± 881.7	2441.8 ± 892.2	0.713
Flow-mediated dilatation				
Response to hyperaemia (%)	5.3 ± 2.3	5.4 ± 2.4	5.1 ± 2.1	0.690
Response to GTN (%)	14.8 ± 5.4	15.2 ± 4.8	14.3 ± 6	0.540
Past medical history				
Myocardial infarction (%)	32 (51)	15 (48)	17 (53)	0.803
Elective PCI (%)	15 (24)	5 (16)	10 (31)	0.237
Positive ETT in those without angiogram/previous MI (%)	11 (18)	7 (23)	4 (13)	0.337
CABG (%)	8 (13)	5 (16)	3 (9)	0.421
Hypertension (%)	31 (49)	15 (48)	16 (50)	0.898
Dyslipidaemia (%)	58 (92)	28 (90)	30 (94)	0.615
Medication				
ACE inhibitors (%)	44 (70)	24 (77)	20 (63)	0.197
ARB (%)	11 (18)	4 (13)	7 (22)	0.348
Beta-blockers (%)	51 (81)	24 (77)	27 (84)	0.482
Calcium-channel blocker (%)	14 (22)	9 (29)	5 (16)	0.508
Statins (%)	57 (91)	30 (97)	27 (84)	0.094
Anti-platelet drugs (%)	62 (98)	30 (97)	32 (100)	0.306
Laboratory measurements				
Creatinine, μmol/L	76.2 ±14.0	78.3 ± 12.1	73.4 ± 15.1	0.504
Urea, mmol/L	5.9 ± 1.2	5.9 ± 0.9	5.7 ± 1.2	0.743
eGFR, mL/min, MDRD	91.7 ± 17.6	92.0 ± 14.8	91.0 ± 20.3	0.580
Fasting insulin, mU/L	17.8 ±10.0	19.5 ± 9.6	15.2 ± 10.0	0.220
Fasting glucose, mmol/L	5.5 ± 0.6	5.5 ± 0.5	5.4 ± 0.5	0.856
FIRI	3.8 ± 2.1	4.2 ± 2.0	3.3 ± 2.2	0.263
HbA1c, mmol/mol	40.2 ± 2.5	39.9 ± 2.6	40.5 ± 2.5	0.279
NTproBNP, pg/mL (median IQR)	877.2 (1166.4)	957.8 (1029.1)	796.5 (1247.0)	0.490
IL-6 pg/mL (median IQR)	0.6 (0.39)	0.6 (0.4)	0.6 (0.4)	0.980
Soluble ST2, ng/mL (median IQR)	18.1 (11.3)	17.2 (11.5)	18.9 (11.9)	0.440
TBARs, µM	3.0 ±1.2	2.9 ±1.5	3.1 ±1.2	0.520

Values are mean ± SD, *n* (%), or median (IQR).

ACE inhibitor, angiotensin-converting enzyme inhibitor; ARB, angiotensin II receptor blockers; CABG, coronary artery bypass grafting; ETT, exercise tolerance test; eGFR, estimated glomerular filtration rate; FIRI, fasting insulin resistance index; GTN, glyceryl trinitrate; HbA1c, glycated haemoglobin; IL-6, interleukin-6; IQR, inter-quartile range; LVM, left ventricular mass; LVMI, left ventricular mass index; MI, myocardial Infarction; MDRD, modification of diet in renal disease; MRI, magnetic resonance imaging; NTproBNP, N-terminal pro B-type natriuretic peptide; PCI, percutaneous coronary intervention; TBARs, thiobarbituric acid reactive substances.

### Primary outcome

**Figure 2:**
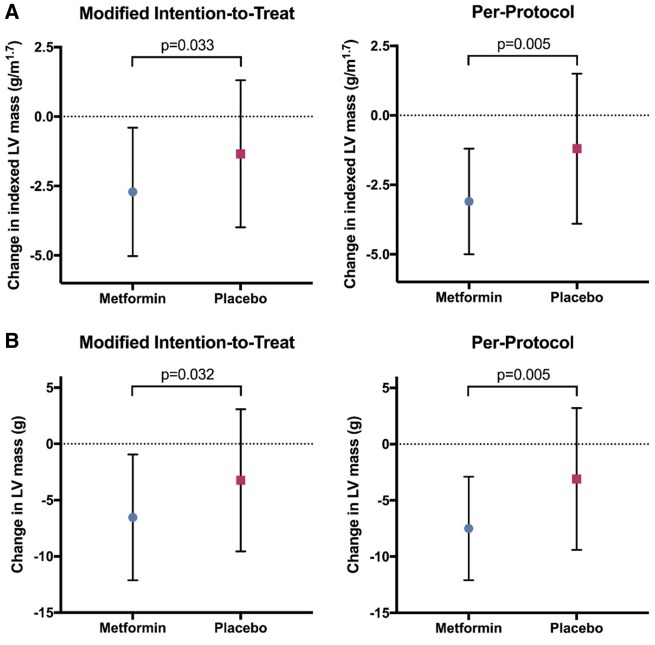
Effect of metformin ton left ventricular mass index and left ventricular mass. (*A*) This graph illustrates the effect of 12 months of metformin or placebo treatment on the left ventricular mass index. Metformin significantly reduced left ventricular mass index after 12 months of therapy compared with placebo (*P* = 0.033 for modified intention-to-treat and *P* = 0.005 for per-protocol analysis). (*B*) This graph illustrates the effect of 12 months of metformin or placebo treatment on the left ventricular mass. Metformin significantly reduced left ventricular mass after 12 months of therapy compared with placebo (*P* = 0.032 for modified intention-to-treat and *P* = 0.005 for per-protocol analysis.

In the mITT analysis, metformin treatment significantly reduced LVMI (change in LVMI: metformin group −2.71 ± 2.31 g/m^1.7^ vs. placebo group −1.34 ± 2.65 g/m^1.7^; *P* = 0.033), leading to an absolute mean difference of −1.37 (95% confidence interval: −2.63 to −0.12). The reduction on LVMI was even greater in the per-protocol population (change in LVMI: metformin group −3.12 ± 1.95 g/m^1.7^ vs. placebo group −1.29 ± 2.67 g/m^1.7^; *P* = 0.005) (*Table 2* and *Figure 2*). Additional sensitivity analysis for the mITT and per-protocol population conducted using a one-way ANCOVA to compare the effectiveness of treatment, adjusting for relevant confounders showed that the change in LVMI observed in metformin group was higher compared with placebo: (i) for mITT arm—estimated marginal means: metformin group −2.8 g/m^1.7^ [95% confidence interval, -3.8 to -1.9] vs. placebo group −1.2 g/m^1.7^ [95% confidence interval, −2.1 to −0.3] and (ii) per-protocol population—estimated marginal means: metformin group −3.3 g/m^1.7^ [95% confidence interval, -4.2 to -2.3] vs. placebo group −1.2 g/m^1.7^ [95% confidence interval, −2.1 to −0.23] and remained statistically significant (*P* = 0.019 for mITT and *P* = 0.004 for per-protocol analysis), suggesting that this finding was robust and not driven by potential relevant baseline characteristics.

**Table 2:** Changes after 12 months of metformin treatment

Outcomes	Modified intention—to—treat analysis				Per-protocol analysis			
	Metformin	Placebo	Difference^a^ (95% CI)	*P*-value	Metformin	Placebo	Difference^a^ (95% CI)	*P*-value
Primary outcome								
LVMI, g/m^1.7^	−2.71 ± 2.31	−1.34 ± 2.66	−1.37 (−2.63 to −0.12)	**0.033**	−3.12 ± 1.95	−1.29 ± 2.67	−1.83 (−3.1 to −0.57)	**0.005**
Key secondary outcomes								
LVM, g	−6.53 ± 5.59	−3.23 ± 6.32	−3.3 (−6.32 to −0.29)	**0.032**	−7.53 ± 4.66	−3.13 ± 6.36	−4.4 (−7.4 to −1.4)	**0.005**
SCAT, %	− 6.74 ± 11.19	−0.27 ± 7.21	−6.47 (−12.06 to −0.89)	**0.024**	−8.84 ± 10.0	−0.28 ± 7.39	−8.56 (−13.95 to −3.16)	**0.003**
Body weight, Kgs	−3.61 ± 4.88	−0.01 ± 3.63	−3.6 (−5.77 to −1.44)	**0.001**	−4.22 ± 4.9	−0.04 ± 3.81	−4.18 (−6.52 to −1.83)	**0.001**
TBARs, µM	−0.26 ± 1.04	0.33 ± 1.14	−0.59 (−1.16 to −0.03)	**0.040**	−0.32 ± 1.1	0.36 ± 1.15	−0.68 (−1.29 to −0.07)	**0.030**
Other outcomes								
SBP, mmHg	−4.81 ± 15.57	4.31 ± 15.26	−9.12 (−16.89 to −1.35)	**0.022**	−4.56 ± 15.82	4.28 ± 15.9	−8.83 (−17.34 to −0.33)	**0.042**

*P*-values in bold indicate *P* < 0.05.

a Absolute mean difference between groups. All values expressed in mean ± SD unless stated.

LVM, left ventricular mass; LVMI, left ventricular mass index; SBP, systolic blood pressure; SCAT, subcutaneous adipose tissue; TBARs, thiobarbituric acid reactive substances.

### Secondary outcomes

There were no significant differences between treatment groups at 12 months for other cardiac secondary endpoints such as LVEF, LVEDV, LVESV, and LVSV except for LVM (Supplementary material online, *Table S1*). Metformin treatment significantly reduced LVM (change in LVM: metformin group −6.53 ± 5.59 g vs. placebo group −3.23 ± 6.32 g; *P* = 0.032) in the mITT analysis, leading to an absolute mean difference of −3.3 (95% confidence interval: −6.32 to −0.29) (*Table 2* and *Figure 2*). The treatment effect on LVM was greater when analysed using the per-protocol population with a highly significant reduction in LVM (change in LVM: metformin group −7.53 ± 4.66 g vs. placebo group −3.13 ± 6.36 g; *P* = 0.005).

Abdominal MRI images were obtained from 61 patients but 13 patients were excluded from this analysis due to inadequate image quality. Hence, 48 (26 and 22 in metformin arm and placebo arm, respectively) and 44 (23 and 21 in metformin arm and placebo arm, respectively) patients were included in the mITT arm and per-protocol population analysis, respectively. Subcutaneous and visceral abdominal tissue (SCAT and VAT) areas were quantified and there was no statistically significant difference between treatment groups at baseline. Treatment with metformin significantly reduced SCAT (mITT: % change in SCAT: metformin group −6.74 ± 11.19 vs. placebo group −0.27 ± 7.21; *P* = 0.024; per-protocol: metformin group −8.84 ± 10 vs. placebo group −0.28 ± 7.39; *P* = 0.003) (*Table 2*) whereas metformin treatment did not have a significant effect on VAT (Supplementary material online, *Table S1*).

We did not observe any effect of metformin treatment on FMD in both mITT and per-protocol analysis (Supplementary material online, *Table S1*).

No significant differences were identified in changes in concentration of NTproBNP, soluble ST2 and IL-6 (Supplementary material online, *Table S1*). However, metformin treatment significantly reduced the concentration of TBARs; a by-product of lipid peroxidation and a marker of oxidative stress (change in TBARs: metformin group −0.26 ± 1.04 µM vs. placebo group 0.33 ± 1.14 µM; *P* = 0.04) in the mITT analysis (*Table 2*).

At baseline, patients in the metformin group had a slightly higher level of FIRI and fasting insulin. There was no significant metformin treatment induced changes in glycaemic parameters such as fasting plasma glucose, fasting insulin, HbA1c, or FIRI but there was a statistically significant reduction in fasting glucose (*P* = 0.009) when analysed in the per-protocol population (Supplementary material online, *Table S1*).

#### Effect of metformin on office blood pressure and weight

Compared with placebo, metformin treatment resulted in a significant reduction of body weight and SBP in the mITT and per-protocol analysis arm of the study (*Table 2* and Supplementary material online, *Figure S2*). These findings on office SBP and weight reduction were corroborated by a subgroup analysis, which showed that reduction of office SBP, and body weight was progressive over the entire duration of study (Supplementary material online, *Figure S3*). No correlation was observed between change in LVM and change in body weight and SBP [Pearson correlation, *r* (61) = 0.07, *P* = 0.569 for change in LVM and change in BP; Pearson correlation, *r* (61) = 0.21, *P* = 0.105 for change in LVM and change in body weight].

### Tolerability and safety of metformin

Study medication was generally well tolerated with no reported cases of lactic acidosis or renal impairment during follow-up. There were 34 AEs and 11 SAEs recorded for the study cohort; 19 AEs and 6 SAEs in the metformin group (*N* = 31); and 15 AEs and 4 SAEs in the placebo group (*N* = 32). Majority of patients in the metformin group experienced gastrointestinal disorders (diarrhoea = 47%; flatulence =26%; abdominal discomfort = 5%) compared with placebo group. Although majority of these AEs were transient and were mild to moderate in severity, study medication was prematurely terminated for five patients (metformin group = 4, due to severe diarrhoea or abdominal discomfort; placebo group = 1, developed T2DM). Dose was reduced to 1 g daily for four patients in the metformin group due to diarrhoea or abdominal discomfort at the higher dose. The mean tolerable dose of metformin was 1610 mg per day in this study. There were no reported CV (myocardial infarction, arrhythmias, stroke, and heart failure) SAEs in the placebo arm, but one participant in the metformin group had a stroke.

## Discussion

The main finding of our study is that a modified-release 2000 mg daily dose of metformin treatment significantly reduced LVMI in patients without T2DM who have CAD, LVH and IR and/or pre-diabetes who were optimally treated with evidence-based therapy. The regression of LVH observed in this study was independent of changes in IR. We also found that metformin reduced measures of obesity, reduced SBP and oxidative stress compared with placebo. All these findings were consistent in both mITT and per-protocol analysis, suggesting a robust beneficial cardio-protective effect of metformin in this group of patients.

The CV benefits of metformin has largely been underpinned by the United Kingdom Prospective Diabetes Study (UKPDS) that reported lower CV mortality and morbidity in patients treated with metformin.^27^ However, recent meta-analysis suggests that the CV effects of metformin could be smaller than that reported by UKPDS; however, this should be interpreted with caution as there has only been a small number of RCTs.^28^ While patients with cardiovascular disease (CVD) with T2DM comorbidity are likely to benefit most from metformin, indications of CV benefit over other diabetes treatments has driven interest in repurposing metformin to treat CVD, irrespective of diabetes status.^21^

To the best of our knowledge, this is the first RCT investigating the effect of metformin on LVH in non-diabetic CAD patients identified to have IR and/or pre-diabetes. Our findings are consistent with experimental animal studies showing that metformin can regress LVH.^18^ With regards to clinical studies, a small (*n* = 40), open-labelled, echocardiographic study reported that 6 months treatment with metformin reduced LVM and relative wall thickness in non-diabetic subjects with metabolic syndrome.^29^ Furthermore, in an echocardiographic sub-study of the GIPS III trial, metformin treatment for 4 months was associated with a marginal, but non-significant reduction of LVMI in non-diabetic subjects who have had a myocardial infarction.^30^ Conversely, a recent network meta-analysis based on only three metformin trials reported minimal beneficial effects of metformin on LVM in subjects with T2DM.^31^ Taking all this together, the data would suggest that metformin might be able to regress LVH.

Left ventricular hypertrophy is regarded as one of the strongest independent predictors of CV outcome and the LIFE study had conclusively shown that LVH regression *per se* reduces future CV events irrespective of BP changes.^32^^,^^33^ However, it remains to be proven on whether metformin-induced LVH regression can deliver the same magnitude of reduction in CV events as the LIFE study since the magnitude of LVH regression was greater in the LIFE study when patients received treatment for at least 4 years.^6^ We believe that a CV outcome trial of metformin among subjects without T2DM is needed to change clinical practice. In this regard, the CV benefits of metformin are currently being tested in the VA IMPACT trial (https://clinicaltrials.gov/ct2/show/NCT02915198), an outcome trial involving close to 8000 patients similarly identified as in MET-REMODEL to have pre-diabetes and established atherosclerotic CV disease including CAD.

There are plausible mechanisms for why metformin produced LVH regression in our study (*Take home figure*). Firstly, metformin could mediate LVH regression through its effect on BP. A recent pooled meta-analysis of RCTs of metformin on BP in patients without T2DM reported that metformin can significantly lower SBP, especially in patients with impaired glucose tolerance or obesity (BMI ≥ 30 kg/m^2^), with a mean reduction of 5 and 3 mmHg, respectively.^34^ The magnitude of BP reduction was similar in our study with a reduction of SBP (4.6 mmHg) in metformin group. A second plausible mechanism for LVH regression may be metformin induced reduction in body weight. In our study, metformin therapy reduced body weight by approximately 4 kg and reduced MRI measured SCAT by 8.8%. Our results are in keeping with the findings of the CAMERA study involving non-diabetic individuals, where metformin significantly reduced all measures of adiposity (body weight, body fat, BMI, waist, circumference) in non-diabetic patients with CAD, with a mean weight loss of 3.2 kg in metformin group.^35–37^ In our study, we only observed a significant effect of metformin on SCAT and not on VAT. The effect of metformin on VAT in non-diabetic individuals has not been studied before. In T2DM patients, metformin has been reported to reduce VAT although this is not a consistent finding.^38^^,^^39^ Hence, more studies are needed to investigate the impact of metformin therapy on abdominal obesity, especially on endogenous fat depots. Thirdly, oxidative stress has been pathophysiologically linked to LVH and in our study, metformin reduced oxidative stress as observed by the reduction of TBARs, a biomarker of oxidative stress.^40–42^ Our findings are in keeping with the study by Esteghamati *et al.*^43^ that reported that metformin is more effective in reducing oxidative stress compared with lifestyle modification alone. Fourthly, metformin could mediate this through its insulin-sensitizing properties.^16^ Insulin resistance is thought to contribute to changes in cardiac tissue seen in LVH.^44^ In this study, metformin treatment reduced fasting blood glucose but only resulted in non-significant marginal reductions in FIRI and HbA1c. Previous studies on metformin in non-diabetic individuals also reported none or modest effects on HbA1c.^35^^,^^45^ We did not find any changes to vascular function (FMD) in this group of patients. It is noteworthy that the effect of metformin on endothelial function as assessed by FMD has not been consistent.^41^^,^^46^ Finally, as suggested by previous studies with animal models of LVH,^18^ it is plausible that activation of AMPK by metformin could have played a role in the regression of LVH. It is worth noting that other mechanisms such as increasing nitric oxide bioavailability, limiting interstitial fibrosis, reducing the deposition of advanced glycation end-products, and inhibiting myocardial cell apoptosis have also been proposed for metformin’s efficacy in reducing cardiac remodelling and hypertrophy.^47^ However, this remains purely speculative and cannot be directly inferred from this clinical study.

**Take home figure:**
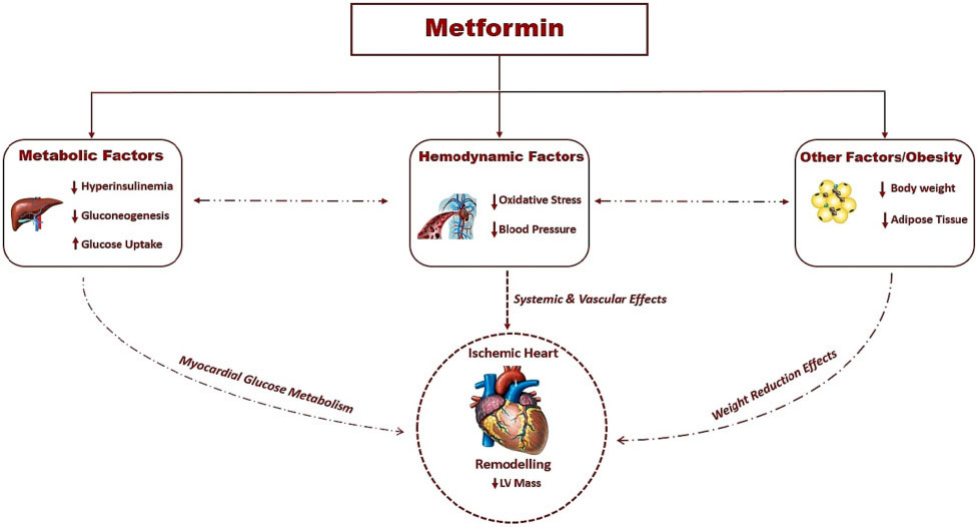
Plausible mechanisms by which metformin regressed left ventricular mass index.

### Limitations of the study

Firstly, this is a single centre study with relatively small number of patients. However, this trial is the largest prospective, adequately powered RCT conducted to date, investigating the efficacy of metformin to regress LVH. Secondly, the study was statistically powered only for a single outcome and not statistically powered to detect changes in other study secondary endpoints. Therefore, inferential between group comparisons for these secondary endpoints are likely to be exploratory rather than definitive. Thirdly, our study was limited to those with CAD and does not address LVH in those without CAD. Fourthly, in this study, we only used one biomarker to measure oxidative stress. We recognize the importance to use more than one criterion to evaluate oxidative stress, as there may be methodological bias associated with different biomarkers for oxidative stress.^48^ Nevertheless, TBARs has been shown as a promising biomarker with prognostic implications, particularly in patients with CAD.^42^ Fifthly, our patient cohort with dysglycaemia have either IR or pre-diabetes or both. We recognize that IR and pre-diabetes may represent different sub-set of populations of patients. Importantly, there was no difference in the proportion of these dysglycaemic states between the two groups. Finally, because of the relatively small sample size, we cannot exclude the possibility that some subtle baseline and demographic differences between the two groups, although not statistically different, might have collectively contributed to our results.

## Conclusion and future directions

In conclusion, this study has shown, for the first time in a RCT that metformin treatment significantly reduced LVMI and LVM compared with placebo in patients with CAD without T2DM. It also improved SBP, reduced oxidative stress and reduced measures of obesity such as body weight and SCAT. Although LVH is a good surrogate marker of CV outcome, we believe that a CV outcome trial of metformin is needed to provide definitive evidence for the cardio-protective role of metformin in non-diabetic CV disease. The results of ongoing CV outcome trials such as the VA IMPACT trial (VA IMPACT NCT02915198) and Glucose Lowering in Non-diabetic hyperglycaemia Trial (GLINT; ISRCTN34875079), will be informative and help provide the needed evidence for recommending metformin in these at risk patients.

## Supplementary Material

ehz203_Supplementary_Material

## Notes

**See page 3418 for the editorial comment on this article (doi: 10.1093/eurheartj/ehz481)**
