# Supplementary material for: A randomized controlled trial of metformin on left ventricular hypertrophy in patients with coronary artery disease without diabetes: the MET-REMODEL trial
Source: Eur Heart J. 2019 Apr 17;40(41):3409–17. doi: 10.1093/eurheartj/ehz203 (PMC6823615; doi:10.1093/eurheartj/ehz203)
Supplement: ehz203_Supplementary_Material [file ehz203_supplementary_material.docx]

**Supplementary Material**

**Table of Contents**

**Section A. Study Inclusion / Exclusion Criteria**

**Section B. Randomization & Masking**

**Section C. MRI Protocols (Cardiac MRI & Abdominal MRI)**

**Section D: Supplementary [S] Figures**

1. Figure S1: Study Design
2. Figure S2: Effect of Metformin on LVMI & LVM
3. Figure S3: Effect of Metformin Treatment on Systolic Blood Pressure and Body Weight over the entire duration of the study

**Section E: Supplementary [S] Table**

Table S1: Changes after 12 months of metformin treatment on other secondary outcomes.

**Section E: References (from 21 - 46)**

**Section A: Study Exclusion Criteria**

3 Left

of CHF

1. Unable to give written informed consent.
2. Diagnosis of Type 1 or 2 diabetes mellitus
3. Left ventricular ejection fraction <45% on screening echocardiography or a diagnosis of heart failure.
4. Contraindications to MRI (pacemakers, claustrophobia, metal implants, history of penetrative eye injury or exposure to metal fragments in eye requiring medical attention)
5. Current malignancies (receiving active treatment) or other life-threatening diseases
6. Renal disease; eGFR ≤ 45
7. Pregnancy and lactating women
8. Participants who have participated in any other clinical trial within the previous 30 days.

4 Contraindications to

**Section B: Randomization & Masking**

Randomization was carried out by Tayside Pharmaceuticals using a validated block randomization method ([www.randomization.com](http://www.randomization.com)). The IMP supply was sequentially numbered and the randomization key held in sealed envelopes by Tayside Pharmaceuticals, Ninewells Pharmacy. The investigating team did not have access to the key until after analysis had taken place. If the study drug was stopped, patients remained in the study in order to perform a “modified intention-to-treat” (mITT) analysis. Participants were allowed to continue all their usual medication throughout the course of the trial.

**Section C: MRI Protocols**

Cardiac MRI Protocol

A stacked acquisition of 2D ECG retrospectively gated segmented breath-hold CINE TrueFISP images were acquired in the short axis orientation from the atrio-ventricular ring to the apex of the left ventricle. Other key imaging parameters were, repetition time (TR) = 3.34 ms; echo time (TE) = 1.46 ms; flip angle (FA) = 50°; field of view 350-450 mm; slice thickness 6mm; inter-slice gap 4mm; resolution 256x256 pixels; and bandwidth of 977 Hz/pixel. Analysis of images was performed offline (Argus Ventricular Function Software, Siemens) by a single observer (S.A.T.) who was blinded to randomized therapy and sequence, for the assessment of ejection fraction, ventricular volumes (EDV, end-systolic volume, stroke volume) and LVM. For each set of images, the observer placed endo-cardial and epi-cardial border contours at end-diastole and end-systole, thus delineating all areas where LV myocardial structure could be observed. Papillary muscle and trabecular structures were included within the blood volume unless indistinguishable from the myocardial wall, in which case they were attributed to the myocardial mass. Each measurement was conducted at least twice for each imaging time-point. The final LVM were indexed to height^1.7^ for each patient (LVMI).

Abdominal MRI Protocol

Subjects were positioned supine inside the magnet and the abdomen was imaged using a 3D ‘DIXON’ volume interpolated breath-hold examination (VIBE) sequence, acquired in a single breath-hold (typically 12 seconds) - providing a series of 64 image slices centred on the L4 vertebra. The imaging parameters were TR = 3.97 ms, TE = 1.26 and 2.49 ms, section thickness 2mm, in-plane pixel resolution 165x320, bandwidth 1040 Hz/pixel, parallel imaging ‘iPAT’ factor 4, and a field-of-view up to 480 mm. The DIXON VIBE sequence was used to obtain ‘fat only’ and ‘water only’ images, as well as images with fat and water signals in/out of phase.

The ‘fat only’ images were analysed using post-processing software ‘Analyze’ (Version 12.0, Mayo Clinic, Rochester, MN, USA). A global signal intensity threshold value was applied to the images in order to separate adipose tissue signals from the background and other tissues present. Manual segmentation methods were then used to remove other signals (e.g. bone marrow) that did not correspond to either visceral or subcutaneous adipose tissue. Images were analysed on a slice-by-slice basis in this manner in order to derive two fat compartments – the sub-cutaneous adipose tissue (SCAT) and visceral adipose tissue (VAT). All volumes were analysed by a single observer who was blinded to randomized therapy and sequence to minimise variation.

Reproducibility and Variability of MRI

For Cardiac MRI: Two measurements of LVM were obtained for every study participant at baseline time-point by a single observer. We elected to use a single observer to undertake all of the segmentation work in order to optimise the reproducibility since it is widely known that inter-observer variation tends to introduce the greatest variability in these measurements. For all of the LVM values we examined every ‘segmentation pair’ closely. By recording the mean LVM in each case, this process lead to improved confidence that the actual LVM values (and changes) were correct and representative of genuine change - i.e. not just measurement error. From these paired baseline measurements of LVM we calculated the intra-class correlation coefficient (ICC) to be 0.98 and we obtained an intra-observer test-retest root-mean-square coefficient of variation (RMS CoV) of 3.77%.

For Abdominal MRI: For abdominal adiposity, repeatability data were not derived specifically for this study cohort.  However, the methods used were identical to those reported by Marzetti et al ^46^ where the top of the L3 intervertebral disc and base of the L5 intervertebral disc were used as anatomical boundaries to determine MRI slice limits for the localised volume assessments of visceral adipose tissue (VAT) and subcutaneous adipose tissue (SCAT).  In the above work, the intra-observer test retest RMS coefficient of variation (CoV) was measured to be 3.47% for VAT and 1.17% for SCAT, and we are confident that similar figures would apply to the cohort studied in this work - since we used the same software and analysis methods

**Section D: Supplementary [S] Figures**


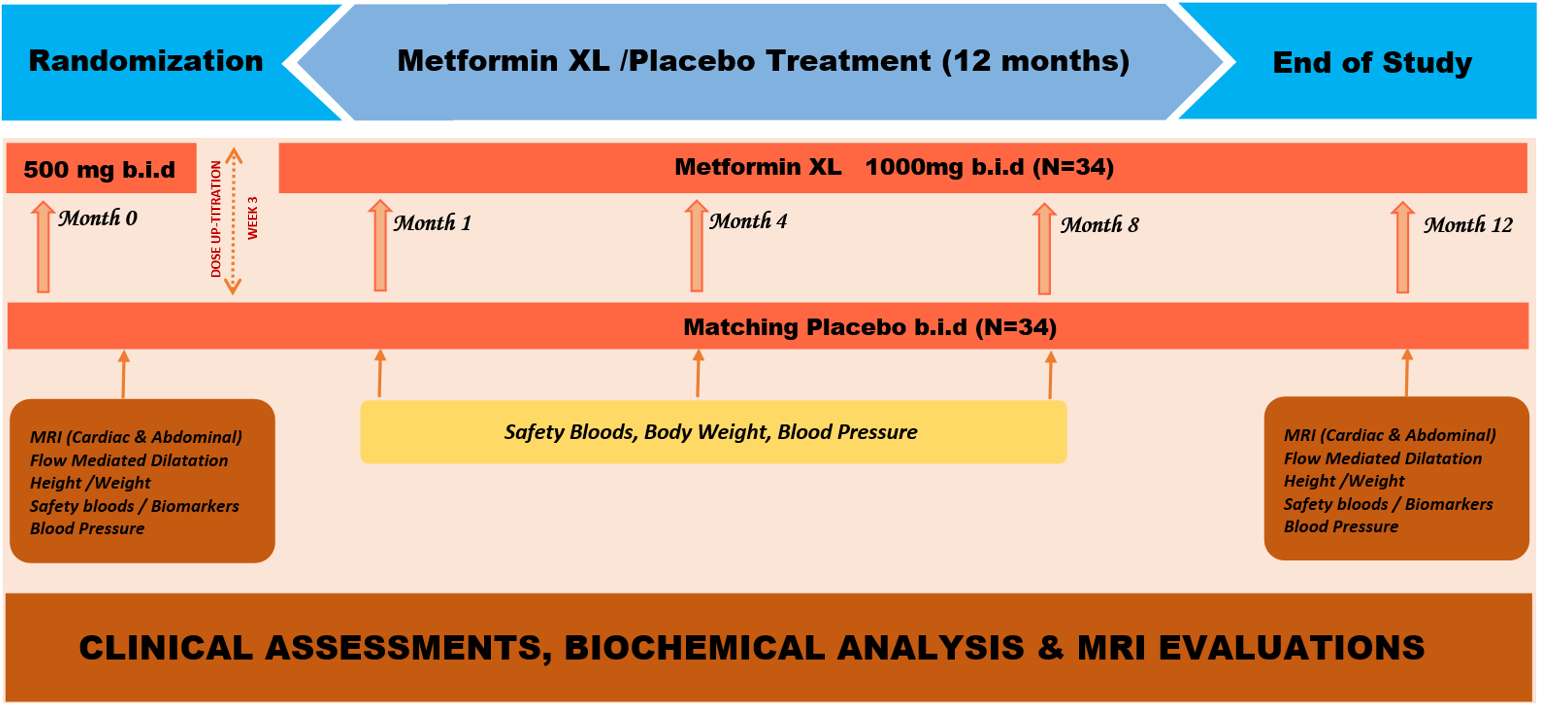


**Figure S1: Study Design**

**Figure S2: Effect of Metformin on Body Weight and Systolic Blood Pressure**

**
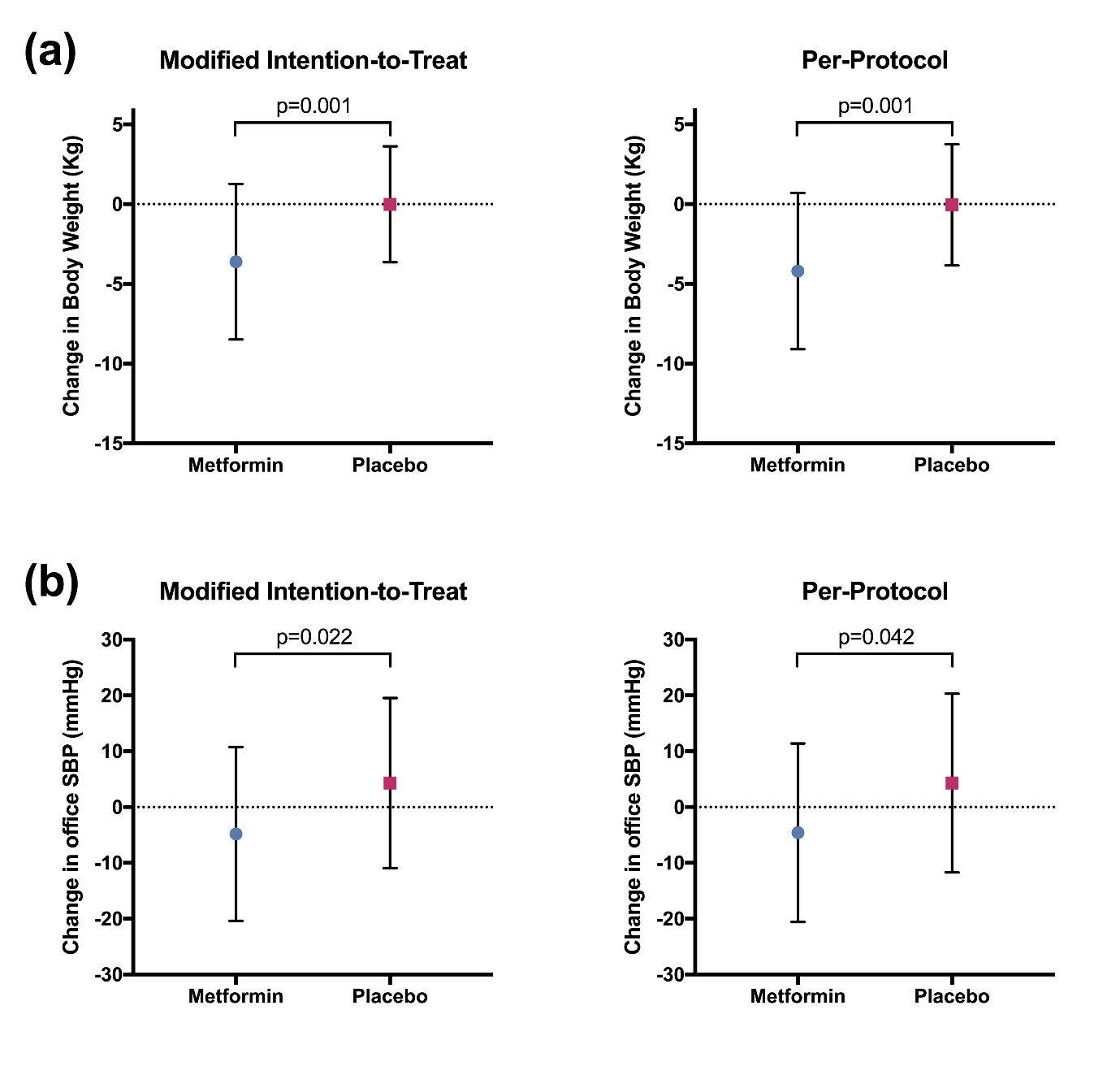
**

1. This graph illustrates the effect of 12 months of metformin or placebo treatment on body weight. Metformin significantly reduced body weight after 12 months of therapy compared with placebo (p = 0.001 for mITT and p=0.001 for per-protocol analysis).
2. This graph illustrates the effect of 12 months of metformin or placebo treatment on systolic blood pressure. Metformin significantly reduced systolic blood pressure after 12 months of therapy compared with placebo (p = 0.022 for mITT and p=0.042 for per-protocol analysis.

**Figure S3: Effect of Metformin on Body Weight and Systolic Blood Pressure over the Entire duration of the Study**


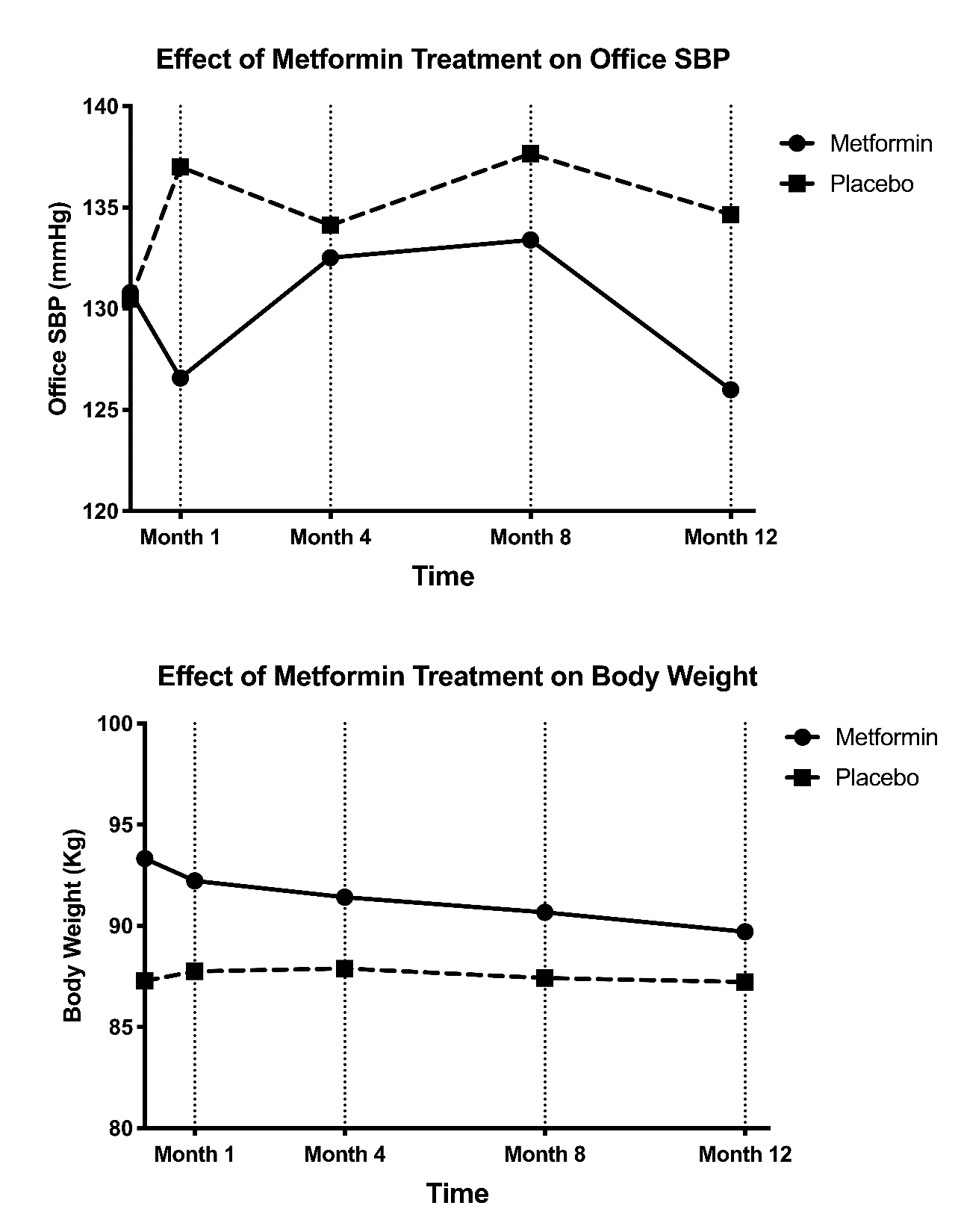


This graph illustrates the effect of 12 months of metformin or placebo treatment on reduction of office SBP and body weight was progressive over the entire duration of study.

**Section E: Supplementary [S] Table**

**Table S1. Changes after 12 months of metformin treatment**

| **Outcomes** | **Modified Intention – to – Treat Analysis** | | | | **Per-Protocol Analysis** | | | |
| --- | --- | --- | --- | --- | --- | --- | --- | --- |
|  | **Metformin** | **Placebo** | **Difference***  **(95% CI)** | **P Value** | **Metformin** | **Placebo** | **Difference***  **(95% CI)** | **P Value** |
| **Other Secondary Outcomes** | | | | | | | | |
| LVEF,% | - 3.58 ± 7.98 | - 3.53 ± 6.58 | -0.05 (-3.73 to 3.63) | 0.980 | - 4.11 ± 8.39 | - 3.90 ± 6.81 | - 0.21 (- 4.30 to 3.88) | 0.918 |
| LVEDV, mls | 2.18 ± 16.91 | 7.05 ± 10.53 | - 4.87 (-11.95 to 2.20) | 0.174 | 0.77 ± 16.24 | 6.68 ± 9.86 | - 5.91 (-13.1 to 1.23) | 0.103 |
| LVESV, mls | 6.53 ± 13.66 | 7.34 ± 9.44 | - 0.81 (-6.71 to 5.10) | 0.784 | 6.97 ± 14.31 | 7.67 ± 9.68 | -0.70 (-7.21 to 5.80) | 0.829 |
| LVSV, mls | - 4.33 ± 15.49 | - 0.29 ± 12.50 | - 4.04 (-11.12 to 3.05) | 0.259 | -6.17 ± 15.14 | - 0.99 ±12.6 | - 5.20 (-12.63 to 2.27) | 0.172 |
| VAT,% | 0.48 ± 15.92 | -1.38 ± 15.37 | 1.86 (-7.28 to 11.0) | 0.684 | -1.07 ± 16.24 | -0.51±15.18 | - 0.56(-10.16 to 9.03) | 0.907 |
| FMD response to hyperemia, % | 0.96 ± 1.10 | 1.11 ± 1.15 | -0.15 (-0.74 to 0.45) | 0.618 | 0.87 ± 1.15 | 1.27 ± 1.17 | - 0.39 (-1.10 to 0.30) | 0.257 |
| FMD response to GTN, % | 0.004 ± 4.24 | 1.30 ± 2.51 | -1.30 (-3.28 to 0.69) | 0.195 | -0.46 ± 4.06 | 1.3 ± 2.56 | -1.77 ( -3.78 to 0.25) | 0.085 |
| IL-6, pg/ml, (median IQR) | 0.44 ± 1.86 | 0.43 ± 1.46 | 0.02(- 0.85 to 0.88) | 0.973 | 0.49 ± 2 | 0.43 ± 1.48 | 0.06(- 0.88 to 1.01) | 0.895 |
| Soluble ST2, ng/ml, | 0.97 ± 4.65 | - 0.18 ± 3.74 | 1.15 (-1.04 to 3.33) | 0.297 | 1.2 ± 4.93 | -0.19 ± 3.8 | 1.39 (-0.98 to 3.76) | 0.244 |
| NTproBNP, pg/ml, (median IQR) | 308.71 ± 1389.84 | 98.10 ± 474.55 | 210.62 ( -326.11 to 747.34) | 0.435 | 375.9 ± 1478.62 | 70.43 ± 457.67 | 305.43 (- 273.46 to 884.31) | 0.295 |
| HbA1c, mmol/mol | -1.48 ± 2.29 | - 0.63 ± 2.13 | - 0.86 (-1.98 to 0.26) | 0.129 | -1.52 ± 2.46 | - 0.69 ±2.23 | - 0.83 (- 2.09 to 0.43) | 0.192 |
| FIRI | - 0.74 ± 2.26 | 0.40 ± 3.33 | -1.14 (-2.59 to 0.31) | 0.120 | -0.87 ± 2.37 | 0.43 ± 3.45 | -1.31 (- 2.91 to 0.29) | 0.106 |
| Fasting Insulin, mU/L | -3.03 ± 10.51 | 0.67 ± 12.45 | -3.70 (- 9.6 to 2.2) | 0.214 | -3.59 ± 11.07 | 0.72 ± 12.9 | - 4.31(-10.83 to 2.21) | 0.189 |
| FBG, mmol/L | - 0.20 ± 0.54 | 0.08 ± 0.72 | -0.28 (- 0.6 to 0.04) | 0.087 | - 0.25 ± 0.56 | 0.17± 0.61 | -0.43 (- 0.74 to - 0.11) | **0.009** |
| **Other Outcomes** | | | | | | | | |
| DBP, mmHg | -2.58 ± 7.72 | - 0.69 ± 8.95 | -1.89 (-6.11 to 2.33) | 0.373 | -2.92 ± 7.7 | - 0.86 ± 9.39 | - 2.06 (- 6.69 to 2.56) | 0.375 |

P-values in bold indicate P<0.05; ***** Absolute mean Difference between groups**.** All values expressed in mean ± SD unless stated.

Abbreviations: DBP, Diastolic Blood Pressure; FBG, fasting blood glucose ; FIRI, fasting insulin resistance index; FMD, Flow Mediated Dilatation; GTN, glyceryl trinitrate; HbA1c, glycated haemoglobin A1c; IL-6, interleukin 6; LVEF, Left Ventricular Ejection Fraction; LVEDV, Left Ventricular End Diastolic Volume; LVESV, Left Ventricular End Systolic Volume; LVSV, Left Ventricular Stroke Volume; NT-proBNP, N-terminal pro B-type natriuretic peptide; SBP: Systolic Blood Pressure ; SCAT, Sub-cutaneous Adipose Tissue; TBARs, Thiobarbituric acid reactive substances ; VAT, Visceral Adipose Tissue.

**Section E: References (from 21- 49)**

21. Rena G, Lang CC. Repurposing Metformin for Cardiovascular Disease. Circulation 2018;**137**(5):422-424.

22. AlZadjali MA, Godfrey V, Khan F, Choy A, Doney AS, Wong AK, Petrie JR, Struthers AD, Lang CC. Insulin resistance is highly prevalent and is associated with reduced exercise tolerance in nondiabetic patients with heart failure. J Am Coll Cardiol 2009;**53**(9):747-53.

23. American Diabetes A. Diagnosis and classification of diabetes mellitus. Diabetes Care 2010;**33 Suppl 1**:S62-9.

24. Chirinos JA, Segers P, De Buyzere ML, Kronmal RA, Raja MW, De Bacquer D, Claessens T, Gillebert TC, St John-Sutton M, Rietzschel ER. Left ventricular mass: allometric scaling, normative values, effect of obesity, and prognostic performance. Hypertension 2010;**56**(1):91-8.

25. Corretti MC, Anderson TJ, Benjamin EJ, Celermajer D, Charbonneau F, Creager MA, Deanfield J, Drexler H, Gerhard-Herman M, Herrington D, Vallance P, Vita J, Vogel R, International Brachial Artery Reactivity Task F. Guidelines for the ultrasound assessment of endothelial-dependent flow-mediated vasodilation of the brachial artery: a report of the International Brachial Artery Reactivity Task Force. J Am Coll Cardiol 2002;**39**(2):257-65.

26. Rekhraj S, Gandy SJ, Szwejkowski BR, Nadir MA, Noman A, Houston JG, Lang CC, George J, Struthers AD. High-dose allopurinol reduces left ventricular mass in patients with ischemic heart disease. J Am Coll Cardiol 2013;**61**(9):926-32.

27. Effect of intensive blood-glucose control with metformin on complications in overweight patients with type 2 diabetes (UKPDS 34). UK Prospective Diabetes Study (UKPDS) Group. Lancet 1998;**352**(9131):854-65.

28. Griffin SJ, Leaver JK, Irving GJ. Impact of metformin on cardiovascular disease: a meta-analysis of randomised trials among people with type 2 diabetes. Diabetologia 2017;**60**(9):1620-1629.

29. Hugo Velázquez, Meaney A, Galeana C, Zempoalteca JC, Gutiérrez-Salmeán G, Nájera N, Ceballos G, Meaney E. Metformin enhances left ventricular function in patients with metabolic syndrome. Rev Colomb Cardiol 2016;**27(1)**:16-25.

30. Al Ali L, Hartman MT, Lexis CP, Hummel YM, Lipsic E, van Melle JP, van Veldhuisen DJ, Voors AA, van der Horst IC, van der Harst P. The Effect of Metformin on Diastolic Function in Patients Presenting with ST-Elevation Myocardial Infarction. PLoS One 2016;**11**(12):e0168340.

31. Ida S, Kaneko R, Murata K. Effects of oral antidiabetic drugs on left ventricular mass in patients with type 2 diabetes mellitus: a network meta-analysis. Cardiovasc Diabetol 2018;**17**(1):129.

32. Ruilope LM, Schmieder RE. Left ventricular hypertrophy and clinical outcomes in hypertensive patients. Am J Hypertens 2008;**21**(5):500-8.

33. Dahlof B, Devereux RB, Kjeldsen SE, Julius S, Beevers G, de Faire U, Fyhrquist F, Ibsen H, Kristiansson K, Lederballe-Pedersen O, Lindholm LH, Nieminen MS, Omvik P, Oparil S, Wedel H, Group LS. Cardiovascular morbidity and mortality in the Losartan Intervention For Endpoint reduction in hypertension study (LIFE): a randomised trial against atenolol. Lancet 2002;**359**(9311):995-1003.

34. Zhou L, Liu H, Wen X, Peng Y, Tian Y, Zhao L. Effects of metformin on blood pressure in nondiabetic patients: a meta-analysis of randomized controlled trials. J Hypertens 2017;**35**(1):18-26.

35. Preiss D, Lloyd SM, Ford I, McMurray JJ, Holman RR, Welsh P, Fisher M, Packard CJ, Sattar N. Metformin for non-diabetic patients with coronary heart disease (the CAMERA study): a randomised controlled trial. Lancet Diabetes Endocrinol 2014;**2**(2):116-24.

36. Knowler WC, Barrett-Connor E, Fowler SE, Hamman RF, Lachin JM, Walker EA, Nathan DM, Diabetes Prevention Program Research G. Reduction in the incidence of type 2 diabetes with lifestyle intervention or metformin. N Engl J Med 2002;**346**(6):393-403.

37. Saenz A, Fernandez-Esteban I, Mataix A, Ausejo M, Roque M, Moher D. Metformin monotherapy for type 2 diabetes mellitus. Cochrane Database Syst Rev 2005(3):CD002966.

38. Golay A. Metformin and body weight. Int J Obes (Lond) 2008;**32**(1):61-72.

39. Jonker JT, Lamb HJ, van der Meer RW, Rijzewijk LJ, Menting LJ, Diamant M, Bax JJ, de Roos A, Romijn JA, Smit JW. Pioglitazone compared with metformin increases pericardial fat volume in patients with type 2 diabetes mellitus. J Clin Endocrinol Metab 2010;**95**(1):456-60.

40. Seddon M, Looi YH, Shah AM. Oxidative stress and redox signalling in cardiac hypertrophy and heart failure. Heart 2007;**93**(8):903-7.

41. Mather KJ, Verma S, Anderson TJ. Improved endothelial function with metformin in type 2 diabetes mellitus. J Am Coll Cardiol 2001;**37**(5):1344-50.

42. Walter MF, Jacob RF, Jeffers B, Ghadanfar MM, Preston GM, Buch J, Mason RP, study P. Serum levels of thiobarbituric acid reactive substances predict cardiovascular events in patients with stable coronary artery disease: a longitudinal analysis of the PREVENT study. J Am Coll Cardiol 2004;**44**(10):1996-2002.

43. Esteghamati A, Eskandari D, Mirmiranpour H, Noshad S, Mousavizadeh M, Hedayati M, Nakhjavani M. Effects of metformin on markers of oxidative stress and antioxidant reserve in patients with newly diagnosed type 2 diabetes: a randomized clinical trial. Clin Nutr 2013;**32**(2):179-85.

44. Paternostro G, Pagano D, Gnecchi-Ruscone T, Bonser RS, Camici PG. Insulin resistance in patients with cardiac hypertrophy. Cardiovasc Res 1999;**42**(1):246-53.

45. Lexis CP, van der Horst IC, Lipsic E, Wieringa WG, de Boer RA, van den Heuvel AF, van der Werf HW, Schurer RA, Pundziute G, Tan ES, Nieuwland W, Willemsen HM, Dorhout B, Molmans BH, van der Horst-Schrivers AN, Wolffenbuttel BH, ter Horst GJ, van Rossum AC, Tijssen JG, Hillege HL, de Smet BJ, van der Harst P, van Veldhuisen DJ, Investigators G-I. Effect of metformin on left ventricular function after acute myocardial infarction in patients without diabetes: the GIPS-III randomized clinical trial. JAMA 2014;**311**(15):1526-35.

46. Naka KK, Papathanassiou K, Bechlioulis A, Pappas K, Kazakos N, Kanioglou C, Kostoula A, Vezyraki P, Makriyiannis D, Tsatsoulis A, Michalis LK. Effects of pioglitazone and metformin on vascular endothelial function in patients with type 2 diabetes treated with sulfonylureas. Diab Vasc Dis Res 2012;**9**(1):52-8.

47. Dziubak A, Wojcicka G, Wojtak A, Beltowski J. Metabolic Effects of Metformin in the Failing Heart. Int J Mol Sci 2018;**19**(10).

48. Marrocco I, Altieri F, Peluso I. Measurement and Clinical Significance of Biomarkers of Oxidative Stress in Humans. Oxid Med Cell Longev 2017;**2017**:6501046.

49. Marzetti M, Brunton T, McCreight L, Pearson E, Docherty S, Gandy SJ. Quantitative MRI evaluation of whole abdomen adipose tissue volumes in healthy volunteers-validation of technique and implications for clinical studies. Br J Radiol 2018;**91**(1087):20180025
